# Commentary: There is no such thing as interoception

**DOI:** 10.3389/fpsyg.2025.1579066

**Published:** 2025-04-23

**Authors:** Jennifer Murphy

**Affiliations:** Department of Psychology, University of Surrey, Guildford, United Kingdom

**Keywords:** interoceptive accuracy, measurement, interoception, interoceptive sensitivity, interoceptive ability

## Introduction

In their article, Schoeller et al. (2025) question whether interoception—namely a unified interoceptive ability—exists. Drawing on evidence that interoceptive accuracy dissociates across domains, the limitations of existing tasks, the unclear mapping of interoceptive accuracy to clinical variables, and the lack of transfer effects in the area of interoceptive training, the authors argue that the existing evidence challenges the notion of a unified interoceptive ability.

## The impact of task limitations and non-interoceptive factors

These arguments are in line with current thinking (Desmedt et al., 2023, 2025) and whilst I agree with many of the arguments posed with respect to the limitations of existing tasks (that are not limited to the Heartbeat Counting Task nor indeed cardiac interoceptive accuracy; see Murphy, 2023) —I believe that these limitations preclude strong conclusions regarding the domain specificity of interoceptive accuracy (and indeed, associations with clinical variables; see for example Adams et al., 2022; Desmedt et al., 2022). Although the authors draw on much evidence suggesting that interoceptive accuracy dissociates across domains (the majority of which has employed tasks that have significant limitations), the authors do not sufficiently address evidence that measures of interoceptive accuracy show poor correspondence even when examined *within* a domain.

We have shown that when pooling data across multiple studies only ~4% of the variance is shared between the Heartbeat Counting Task and Heartbeat Detection Task (Hickman et al., 2020). Even where similar Heartbeat Detection variants are compared, for example comparing two methods that require matching of an external stimulus (e.g., a tone) to one's heartbeat, these measures often only show moderate correspondence (~35% variance shared; Brener et al., 1993; Brener and Ring, 2016). The exception to this low-moderate correspondence occurs only when Heartbeat Detection variants are extremely well-matched; for example, good correspondence is often observed when comparing tasks that present tones at similar delays following the hearts R-wave and use similar analyses strategies that infer accuracy from the consistency of participants' selected delays (up to 52% variance shared between tasks; Brener et al., 1993; Brener and Ring, 2016).

Where evidence suggests that almost 25% of the variance in Heartbeat Detection can be explained by performance in a well-matched, but purely exteroceptive task (Knapp et al., 1997)—an amount not far off that explained by performance across two similarly, albeit not perfectly, matched cardiac interoceptive accuracy tasks—it is perhaps unsurprising that interoceptive accuracy tasks show poor correspondence when compared across domains. Indeed, interoceptive accuracy tasks across domains vary greatly in their task format, as well as the demands they make on non-interoceptive processes (e.g., multisensory integration, sustained attention, working memory etc.; for discussion see Brewer et al., 2021), which may prevent detection of associations between interoceptive accuracy tasks across domains if they do in fact exist. Consistent with this possibility, when task formats are better matched, evidence does suggest the possibility of at least some correspondence across interoceptive domains—notably cardiac and gastric interoceptive accuracy (~25% of the variance shared; Whitehead and Drescher, 1980). The same is true for the examination of transfer effects in the area of interoceptive training—if the training improves non-interoceptive factors that contribute toward performance on a task in one domain, but not the non-interoceptive factors that contribute toward performance in a different domain—one may erroneously conclude that interoceptive accuracy is not a domain general ability.

## Discussion

Whilst it is entirely possible that a unitary interoceptive ability may not exist, and such findings would be consistent with some theory and evidence (e.g., Stephani et al., 2011; Khalsa et al., 2018; but see Kwon et al., 2025; for discussion see Brewer et al., 2021), confirming this requires greater consideration of the non-interoceptive factors that may contribute toward performance on tests of interoceptive accuracy and attempts to match tasks across domains. At a minimum, the contribution of non-interoceptive processes should be established using a well-matched control task. Although such work may be challenging, in light of evidence that tasks within a domain are poorly related, such work is essential before we can make strong conclusions regarding domain specificity. Whilst we may need to be cautious about generalizing findings from one domain to another at this time, it is too early to conclude that “there is no such thing as interoception.”
